# Key recommendations to strengthen public-private partnership for adolescent health in resource constrained settings: Formative qualitative inquiry in Mongolia, Myanmar and the Philippines.

**DOI:** 10.1016/j.lanwpc.2021.100242

**Published:** 2021-08-05

**Authors:** Peter S Azzopardi, Julie Hennegan, Shirley Mark Prabhu, Bolorchimeg Dagva, Mx Mar Balibago, Pa Pa Win Htin, Zay Yar Swe, Elissa C Kennedy

**Affiliations:** 1 Global Adolescent Health Group, Maternal Child and Adolescent Health Program, Burnet Institute, Melbourne, Australia; 2 Adolescent health and wellbeing program, Aboriginal Health Equity Theme, South Australian Health and Medical Research Institute, Adelaide, Australia; 3 Department of Paediatrics, School of Medicine Dentistry and Health Sciences, University of Melbourne, Melbourne Australia; 4 Melbourne School of Population and Global Health, University of Melbourne; 5 Adolescent Health, Mental Health and HIV Specialist, UNICEF Middle East and North Africa Regional Office, Amman, Jordan (formerly UNICEF East Asia and Pacific Regional Office); 6 Adolescent health and HIV/AIDS Specialist, UNICEF Mongolia; 7 Adolescent health and HIV/AIDS Specialist, UNICEF Philippines; 8 Former HIV/AIDS Specialist, UNICEF Myanmar; 9 Myanmar country program, International Development Discipline, Burnet Institute, Yangon, Myanmar; 10 School of Public Health and Preventive Medicine, Monash University, Melbourne, Australia

## Abstract

**Background:**

Public health services can be inaccessible for adolescents. The private sector provides many services, but often in parallel to the public sector. This study aimed to understand current private sector engagement in adolescent health service delivery and develop recommendations to strengthen partnerships.

**Methods:**

The study focussed on Mongolia, Myanmar and the Philippines. An initial participatory workshop in each country was followed by semi-structured key-informant interviews (32 in total) with public and private sector actors and adolescents to explore: perceptions of the public and private sectors, strengths and challenges, existing models of partnership, and insights for successful public-private partnership (PPP). Interview transcripts were analysed thematically, with findings and recommendations verified through a second workshop in Mongolia and the Philippines.

**Findings:**

The private sector already plays a significant role in adolescent health care, and stakeholders reported a genuine willingness for partnership. Strengthened PPP was identified as necessary to improve service accessibility and quality for adolescents, unburden the public sector and introduce new technologies, with advantages for the private sector including improved access to training and resources, and an enhanced public image. Recommendations for strengthened PPP included the need to establish the foundations for partnership, clearly define roles and co-ordinate stakeholders, ensure capacity and sustainability, and monitor and evaluate efforts.

**Interpretation:**

This is the first comprehensive study of public-private partnership for adolescent health in the Asia Pacific region. It identifies stakeholders are willing for stronger partnerships and the benefits this partnership will bring. We define eight key recommendations to enable this partnership across sectors.

## 1 Introduction

Health services are a key platform for optimising adolescent health, [1] yet adolescents experience significant barriers accessing public health services- perhaps more than at any other life stage- including barriers relating to service availability and quality. [2] This appears particularly so for the Asia Pacific region, [3], [4], [5], [6], [7] home to almost 1 billion adolescents and adolescents and young people aged 10-24 years that experience a large and unshifting burden of preventable ill health. [8,9] Countries in this region have amongst the lowest index of Healthcare Access and Quality globally, [10] and whilst data specific to adolescents are sparse, demand satisfied with modern contraception amongst 15-24 year old females (a proxy indicator of health service access and quality) is amongst the lowest globally in Pakistan (32%), Nepal (37%), the Philippines (44%) and Papua New Guinea (45%). [9] This may, in part, reflect low levels of public funding on health: Cambodia, Indonesia, Lao PDR, Myanmar and the Philippines spend less than 1% of Gross Domestic Product on health (global average is approximately 6%). [11]

Given the constraints of public health systems, there is increasing interest in engaging the private health sector to ensure universal health coverage. [12] In the South East Asian region, private health expenditure is the highest of any region globally (48% of total health expenditure, global average 41%), with private health expenditure particularly high in the Philippines and Myanmar (>65%). [13] There is some evidence from this region that adolescents already seek services and advice around sexual and reproductive health (SRH) from the private sector (which includes private clinics, pharmacies and non-government organisations) [14] as these may provide less judgmental care with greater privacy, more convenient locations and opening hours, and with less stigma than public-sector services. [7,15,16]

As such, the critical question is not *if* the private sector should be engaged in adolescent health, but rather, *how*? Most health systems in the Asia Pacific region appear to position the public and private sectors in parallel, and sometimes in competition, but rarely in partnership. Our review of the available literature found no published studies of private sector engagement or partnership specific to adolescent health in the Asia Pacific (see research in context and Appendix 1). There is some evidence for this region in the context of tuberculosis control that indicates private providers see value in engaging with the public sector (and vice versa), with perceived benefits related to knowledge exchange, greater access to resources and assistance to address public health needs. [17] National health policies across the region also identify the need for strengthened private sector engagement, however few articulate how the private sector could be engaged. [18] This study aims to define how the private sector can be better engaged in partnership with the public sector to strengthen health service delivery for adolescents.

## 2 Methods

This study primarily used qualitative enquiry to explore perspectives around private sector engagement and what is required to strengthen partnerships. This study focussed on three resource-constrained countries (Mongolia, Myanmar and the Philippines) in the Asia Pacific, [19] selected by the UNICEF East Asia and Pacific regional office based on their interest in advancing public private partnership within their adolescent health programming; this ensured stakeholders were likely to engage in this research but also that research findings would likely translate to action. These three countries also represent substantial diversity in adolescent population, adolescent health needs, quality of health care and investments in the public and private sectors (Table 1).

**Table 1:** Key indicators of adolescent health and the health system in focal countries

	Global		Mongolia		Myanmar		Philippines	
	*female*	*male*	*female*	*male*	*female*	*male*	*female*	*male*
**Adolescent health indicators**								
*Adolescent population, 10-24 y*	1,801,300,000		708,160		15,009,832		30,160,568	
*Pattern of adolescent health needs*			Injury		Multi-burden		Multi-burden	
*Total disease burden (DALYs/ 100), 10-24 y*	13.2	14.8	11.0	16.5	11.0	16.2	11.7	14.8
*Group 1 burden (DALYs/100), 10-24y*	3.9	3.2	1.8	1.8	2.9	3.7	2.6	2.8
*Injury burden (DALYs/100, 10-24 y*	1.3	3.9	1.5	4.9	1.2	4.0	1.0	3.5
*NCD burden (DALYs/100), 10-24 y*	8.0	7.8	7.8	9.8	6.9	8.5	8.1	8.6
*Tobacco smoking (%), 10-24 y*	2.8	12	3.7	22.6	0.5	9.5	2.9	19.5
*Binge alcohol drinking (%), 15-19y*	9.6	14.4	28.3	23.3	1.1	9.1	8.1	24.7
*Overweight and obesity (%), 10-24y*	18.2	17.8	21.3	18.4	15.8	9.9	12.5	12.9
*Anaemia (%), 10-24y*	27.9	20.1	23.2	25.4	21.8	15.8	12.7	5.5
*Completion secondary edu. (%), 20-24y*	53.3	52.6	86.2	74.8	41.5	34.6	66.9	50.3
*NEET (%), 15-24y*	32.8	10.0	22.4	18.7	25.2	11.2	29.2	15.5
*Child marriage <18y (%), 20-24y*	22.8		5.2		16.0		15.0	
*Adolescent fertility (per 1000), 15-19y*	41.2		15.5		15.5		66.3	
**Health system indicators**								
Quality index (0-100)*			53		42		51	
*Demand contraception met (%), 15-24y*	66.6		65.0		79.3		44.1	
Government expenditure on health (% GDP)**	6		2		1		1	
Government expenditure on health (% total health expenditure)**	52		59		15		33	
Domestic Private Health Expenditure (% total health expenditure)**	41		36		76		67	
*Private sector addressed in national health strategy*	-		Yes- specific roles & actions		Yes- specific roles & actions		Yes- specific legislation	
*Private sector addressed in adolescent health policy/ strategy*	-		No		Need identified		Need identified	

This table summarises key adolescent health needs and the health system for Mongolia, Myanmar and the Philippines, with global estimates provided as a comparator where possible. All adolescent health data are sourced from Azzopardi et al, Lancet (2019). [9] Data on HAQ (*) sourced form Fullman et al, Lancet (2018), [10] with data on expenditure (**) sourced from WHO's Global Health Expenditure database for 2018.This table summarises key adolescent health needs and the health system for Mongolia, Myanmar and the Philippines, with global estimates provided as a comparator where possible. All adolescent health data are sourced from Azzopardi et al, Lancet (2019). [9] Data on HAQ (*) sourced form Fullman et al, Lancet (2018), [10] with data on expenditure (**) sourced from WHO's Global Health Expenditure database for 2018.This table summarises key adolescent health needs and the health system for Mongolia, Myanmar and the Philippines, with global estimates provided as a comparator where possible. All adolescent health data are sourced from Azzopardi et al, Lancet (2019). [9] Data on HAQ (*) sourced form Fullman et al, Lancet (2018), [10] with data on expenditure (**) sourced from WHO's Global Health Expenditure database for 2018.

In each country, a workshop was first held in the capital city to introduce the study, seek stakeholder support, explore public-private partnership broadly, and identify participants for key informant interviews (detailed further in Appendix 2). In brief, workshops (of 30 – 40 participants) included a broad range of stakeholders encompassing government, health care providers from both the public and private sectors, NGOs, relevant UN agencies, faith-based organisations, and youth advocates (aged >16 years so they could provide their own consent for participation). Workshop participants representing these stakeholder groups were identified by the UNICEF country office and local implementation partners (Graduate School of the Mongolian National University of Medical Science, Council for the Welfare of Children in the Philippines, and Burnet Institute Myanmar Research office) in consultation with PA (research lead, a male paediatrician and adolescent health researcher) with the aim of having diverse representation across all these stakeholder groups. Participants were mostly based in the national capital but included regional representation to highlight existing models of public private partnership. The workshop discussions were captured in notes.

Workshops were followed by key informant interviews (KIIs) with selected workshop participants (identified in consultation with UNICEF) as well as other relevant stakeholders identified during the interviews. Ten interviews were conducted in Myanmar, 10 in Mongolia and 12 in the Philippines (two additional stakeholders were recruited to explore identified models of public private partnership in more depth) which included a total of 8 participants from the public sector (4 providers and 4 government), 3 youth advocates and 21 participants from the private sector (6 private providers including doctors, nurses and pharmacists, 6 NGOs, 5 UN agencies and 4 private corporations). Interviews explored the current role of the private sector, advantages and challenges of private and public sector partnership in providing health care, existing models of private sector engagement, and what was required to enable this (topic guide presented in Appendix 3). An overarching ethics approval for interviews was obtained from Alfred Hospital Research Ethics Committee (540/19) with the Ministries of Health in each country providing a letter of support. Additional in-country ethics was recommended and sought in Myanmar through Institutional Review Board, Department of Medical Research (ethics/DMR/2020/008). Workshop participants verbally consented to participate, with formal written consent sought from interview participants.

Interviews were conducted in person by PA in Mongolia (week of the 18^th^ November 2019) and Philippines (week of the 10^th^ February 2020) at participant nominated locations (typically their office), and with the aid of a trained interpreter. In Myanmar the interviews were conducted by an in-country research team (led by ZYS) in Burmese between March- April 2020 (several weeks after the workshop, awaiting local ethical clearance); 9 were in person and one using videoconference given the evolving COVID pandemic. All interviews were audio recorded, transcribed verbatim and translated; the quality of transcription and translation were verified against the audio recordings for Mongolia and the Philippines, with the interview transcripts and translations in Myanmar cross-checked by two bilingual researchers to ensure accuracy.

Based on the initial workshop and project aims, a thematic analysis was undertaken. [20] Following familiarisation, PA used NVivo 12 to code interview transcripts deductively under each project aim to describe: current private sector activities, strengths and challenges of public-private engagement, and existing partnership models. Recommendations for successful PPP were coded inductively, and themes developed. Themes were reviewed through meetings with JH who coded a subset (10%) of transcripts for validation. Workshop notes were also reviewed for any additional themes; workshop notes were particularly useful for mapping existing models of PPP.

Preliminary findings of the analysis were circulated to all workshop and interview participants, as well as additional stakeholders identified by UNICEF. Participants and stakeholders were invited to a series of virtual workshops in February 2021 for those in Mongolia (52 participants) and the Philippines (67 participants); we were unable to hold the workshop as planned in Myanmar due to the coup d’état. Findings were discussed for participant validation. In three groups, participants refined final recommendations to strengthen PPP by considering the issues of adolescent pregnancy, mental health, and obesity.

### 2.1 Role of funding source

This study was funded by UNICEF East Asia and Pacific Regional Office (EAPRO). UNICEF EAPRO helped to conceptualise the study and supported implementation, including the choice of focal countries for this work. UNICEF EAPRO were not directly involved in data collection, analysis or drafting the key recommendations. All authors had access to data in the study, approved the final manuscript and were responsible for the decision to submit the manuscript.

## 3 Results

Key findings related to current private sector engagement in adolescent heath are presented, including perceptions of the private sector, comparative quality of the public and private sectors, and training and regulation across these sectors with respect to adolescent health. Existing partnership models are discussed as are the strengths and challenges of public-private partnership. Key recommendations for strengthened partnership which were validated with participants are then presented.

### 3.1 Current private sector engagement in adolescent health

#### 3.1.1 Perceptions of the private sector

Participants described private sector engagement in adolescent health in all countries. In Myanmar, the private sector mostly included private clinical services (including pharmacies) as well as projects led by non-government organizations (NGOs). In Mongolia, the private sector included private clinical services as well as private companies through corporate social responsibility. Similarly in the Philippines the private sector was recognised as providing a large private clinical sector and some engagement with health services through corporate social responsibility, but there was also a broader recognition of the additional services that private providers may provide for adolescent health in supporting clinical training and access to health information and commodities for adolescents, particularly relating to contraception. Of note, in contrast to the formal definition of the private sector (which includes both for profit and not for profit sectors), most participants identified the private sector to be for-profit, with uncertainty around how UN agencies and NGOs should be classified.

#### 3.1.2 Quality of public and private sector services

In discussing public and private sector health care services, participants highlighted that each private sector had distinct strengths, and areas where they may respectively provide a better quality of service. Participants highlighted that the private sector provides a narrower set of services, but that these services are more aligned with adolescent health needs (or consumer demand), and described services as typically more welcoming and friendly. By contrast, the public sector was described as having the resources and infrastructure to provide a more comprehensive package of services that may meet the broader needs of adolescents.

*There are aspects (of adolescent health) that can be better managed in public hospital. It's because we have all the services in our hospital. We have both adult and paediatric specialists, we have the supportive allied, medical services, we have specialist providers. (Philippines, public and private provider)*

*The quality of the private sector can be better than the public sector. Public sector is more time consuming. Communication is one of the problems for public sector as they cannot consult with the doctor freely because of crowded area. And so, the private sector gives more confidentiality. Patients are more satisfied with the private sectors (Myanmar, private provider)*

*If you pay .. service will be much better than getting free public services. That is very common understanding. That's why people like to have paid services at private clinics. Usually public hospitals have lots of patient loads, customers wait too much and waiting line is way too long. So, it's better to get paid services from the private hospitals. (Mongolia, young person)*

#### 3.1.3 Training and regulation to meet adolescent health needs

Participants described varied opportunities for adolescent health training and professional development, that were quite different across sectors. In all countries there had been efforts to train clinicians around adolescent health, but efforts driven by government typically only targeted the public sector. As a result, many private providers developed their own training and resources, however the quality of these was unclear and many identified challenges in identifying relevant training resources more broadly for adolescent health. Regulation was also quite different across the sectors, with the private sector appearing to be less regulated than the public, but this was not always seen as a disadvantage as it enabled the private sector to be more responsive and agile. However, some private sector providers identified difficulty in aligning their service with policy around adolescent health:

*I looked for regulations and policy documents ... I couldn't find any policy or government documentation (Mongolia, private provider)*

### 3.2 Existing examples of partnership models

Despite enthusiasm for PPP there were few examples of partnership models specific to adolescents. In Myanmar, the most notable has been a previous partnership between Government and a private company to provide sanitary pads in public schools. In Mongolia, models of PPP have been driven by private companies as part of their corporate social responsibility and included provision of financial but also technical resources; notable examples were the partnership between mining and the UN to support adolescent reproductive health, [21] and the partnership between telecommunications, an international NGO and government to support a children's helpline. [22] Similarly in the Philippines there are examples of partnership framed around corporate social responsibility. For example, a partnership between government, NGO and a private mall has provided an adolescent health clinic (the mall provides a site and basic infrastructure support). Private providers in the Philippines have also supported specialist adolescent training for public providers.

### 3.3 Strengths and challenges of public-private partnership

#### 3.3.1 Overarching challenges

There were a number of overarching challenges identified in engaging the private sector for adolescent health, particularly so by stakeholders from the public sector. A key issue raised was potential conflict of interest with the private sector focussed on revenue generation, rather than health care. There was concern around the potential lack of comprehensive care, and also a lack of long-term commitment to programs. There was also concern around how activities in the private sector may be monitored or regulated.

#### 3.3.2 Advantages for adolescents

Participants identified that the private sector may help respond to particularly sensitive issues (particularly SRH and mental health). A common theme was that the private sector provides services that are confidential – because there are less people in the waiting room, but also in some instances because private providers do not require or enforce parental consent, so that adolescents can attend on their own

*.. [adolescents] want to hide their sexual health from their parents. They visit on their own to the private clinics (Mongolia, private clinician)*

*Adolescents prefer the private sector mainly for SRH problems…public sector provides mainly for married (Myanmar, NGO)*

The private sector was also perceived as enabling improved and reliable access to commodities, such as contraception. The improved physical facilities and environments of private clinics were identified, particularly in Mongolia. Some identified the private sector as being ‘closer’ to adolescents, and therefore in a better position to advocate for their needs. Participants across countries also identified that the private sector can provide services that are more accessible, efficient and less judgemental.

*The private sectors provide contraceptive services and STI/HIV services to adolescents. They do not take a history to see if they are married or unmarried and they provide services easily (Myanmar, Government).*

*They (the private sector) would provide only essential services or things that they need, and to be done quickly (Mongolia, Clinician)*

Private sector engagement may also enable service access through provision of services in schools, workplaces other settings which adolescents frequent.

*Adolescent clubs and workshops, reading corners and sports (clubs) should be involved in adolescent health (Myanmar, young person).*

#### 3.3.4 Advantages for the public sector

Participants identified private sector engagement as ‘filling the gap in public care’. For the public sector, advantages of private sector engagement identified by participants included increased capacity (human, financial) to respond to adolescent health needs, and also new skills and technologies that the private sector may bring. In some instances, the private sector may uniquely be able to respond to adolescent health needs; in the Philippines several participants spoke about the Temporary Restraining Order that has prohibited the public sector (but not necessarily the private) from providing the contraceptive pill and implants. It was also recognised that the private sector already has established engagement in adolescent health care, and therefore engagement across sectors is essential to providing co-ordinated and efficient care.

Participants from all countries also identified that the private sector can play a particular role in preventative interventions to compliment the public sector's focus on curative health care that responds to disease and injury.

*… private sector can more contribute to healthy lifestyle and healthy behaviour. They support physical fitness and sport products for those activities and harm reduction projects. Government will focus on policy revision, implementation and monitoring… (Myanmar, NGO manager).*

Strengthened private sector engagement was also identified as important to addressing emerging issues (such as mental health and obesity). In part this reflected the broad composition and skills of the private sector, but also agility and increased access to new technologies.

#### 3.3.5 Advantages for the private sector

For the private sector, strengthened linkage with the public health system was seen to improve their public image. This was particularly so for large corporations where investment in adolescent health as part of their corporate social responsibility was seen as a strategy to increase market share and differentiate themselves from competitors. For private clinicians, stronger engagement with the public sector was seen as a means of recognising their role and contribution to adolescent health. Private providers noted that partnership with the public sector may improve the quality of care they provide through linkage with broader services, but also access to training and guidelines.

### 3.6 Recommendations for effective public private partnership for adolescent health

All stakeholders - including adolescents themselves - expressed a genuine willingness for closer public- private collaboration around adolescent health.

*Private sector is an essential sector for youths and adolescent health for our country and developing countries. (Myanmar, young person)*

*Really hope that this [research] can help engage the private sector (with public), help make it happen (Mongolia, Private provider)*

Box 1 shows the key recommendations to strengthen PPP for adolescent health. Workshop participants in Mongolia and Philippines found these recommendations useful for working through models of PPP with respect to key adolescent health issues. These recommendations can be summarised as establishing the foundations for partnership (recommendations 1-2), defining roles and co-ordination (recommendations 3-4), ensuring capacity and sustainability (recommendations 5-6), and monitoring and evaluation (recommendations 7-8).

### 3.7 Establishing foundations for partnership

Recognising adolescent health as a priority was identified as an important foundation to strengthened partnership, or indeed any action around adolescent health.

*Adolescent health is a pressing matter for our country. In general, it is being neglected between family hospitals and educational institutions and (has received) insufficient coverage (Mongolia, Government)*

High quality data - on adolescent health needs but also on sectors, their current activities and their capacity to respond - was identified as important to informing effective partnerships for adolescent health. These data were also considered essential to informing a goal/purpose that aligns with needs and/or policy. Having clear incentives for all involved was also identified as foundational. Of note, these incentives may be different for different sectors, but nonetheless need to be articulated.

*…health care providers tend to think too much what's best for the population, while some private companies are what's in there for us. For a private provider, what are they getting out of this? (Philippines, public and private provider)*

### 3.8 Defining roles and co-ordination of partners

Having clear roles for all stakeholders (including adolescents) that builds on strengths was recommended, as was strong leadership. Many participants – especially in Myanmar and in Mongolia to a large extent - thought that such partnership should be led by government. However, there was also the recommendation that NGOs may play an important convening and leadership role.

*The NGOs/CSOs are like magnets. They are closest to the people, they make significant demands to the public sector and well (without them) conversation between the public and private sector rarely happens…(Philippines, private sector)*

There was a recognised need for policy and legislation that enables the private sector to access the same training, licensing, accreditation and professional development, as well as access to health insurance payments, as public sector providers. However, there was also caution that any new legislation should not be too onerous so as to hamper the agility and responsiveness of the private sector, identified as key benefits of the private sector.

Coordination of partners was strongly recommended, especially as many identified this as a reason why previous efforts had not worked well. Internal coordination should include regular communication but also integration of systems across sectors; one clinician gave the example of difficulties in requesting diagnostic services across the public and private sectors. There were also recommendations that external communication should be co-ordinated, recognising the role of all stakeholders and also targeting adolescents.

*.. when the government authority (talks about our partnership project) ..sometimes they miss out our names. So, we talk to them…constant communications with government authority keep minding them we are still here. (Mongolia, private sector)*

*I think, if they (adolescents) need help, they just google it and the first result of the search is our center Facebook (Mongolia, Private non-profit provider)*

### 3.9 Ensuring capacity and sustainability

A core set of recommendations related to ensuring capacity and sustainability. Indeed, it was recognised that perhaps all sectors require capacity development for adolescent health, and that this capacity development should be standardised across sectors. There were important recommendations around ensuring financial sustainability, with a key recommendation being the need for long term partnership.

*Deeper and longer partnership, those that benefit both sides (Philippines, UN agency)*

### 3.10 Monitoring and evaluation

Monitoring and evaluation (M&E) was also identified as key to successful public-private partnership, especially given many identified a lack of M&E may be the reason why previous efforts have not worked well. Monitoring was identified to be important to ensure that all stakeholders delivered what they agreed to do, an important aspect of building trust between sectors. Evaluation was identified as important to maintaining engagement and interest of stakeholders, but also relevance of the partnership to adolescent health needs.

*There will be mutual trust between public and private if there is accreditation system and guidelines are the same in both sectors (Myanmar, private provider)*

*(with respect to previous engagement in PPP) I was really wondering how useful it was. Was there a monitoring? Was there an evaluation? Where it is no? How it is being used? And the other thing is, since the time we made it, have new issues have come up? (Philippines, private provider*).

## 4 Discussion

The private sector already plays a key role in adolescent health care. Engaging with providers across the public and private sectors, as well as from government, key agencies and adolescents themselves in Mongolia, Myanmar and the Philippines we found a genuine willingness for stronger partnership and identified recommendations to achieve this. Strengthened PPP was identified to have many advantages for adolescents, particularly improved service accessibility and quality; this advantage of PPP is of particular relevance to adolescent health, as adolescents are among the most underserved by public health services. Stronger partnership may unburden the public sector and introduce new technologies, with advantages for the private sector including improved access to training and resources and an enhanced public image. Strengthened PPP was identified to play a particular important role in addressing emerging issues, sensitive issues, and perhaps playing a stronger role in preventative and promotive intervention to complement the public sector's focus on therapeutic care. These are particularly critical for adolescent health, as embarrassment or fear of stigma associated with sensitive health issues, such as sexual and reproductive health, are important barriers to seeking public services. Adolescence is also a key age group for health promotion and preventive services targeting risk factors that typically arise during this life stage, however these interventions are not typically the focus of public services. Key recommendations for strengthened PPP included the need to establish the foundations for partnership, definition of roles and co-ordination of sectors, ensuring capacity and sustainability, and monitoring and evaluation.

What is clear is that the concept of PPP for adolescent health is not new and there are several good examples, although these remain to be evaluated; it appeared that this lack of evaluation may have contributed to these partnerships not being sustained or taken to scale. Of interest are the examples of PPP from Mongolia and the Philippines that have been driven to a large extent by private companies through their commitments to corporate social responsibility. [23,24] Whilst these projects filled a gap in existing services, they also had clearly articulated benefits for private sector actors directly (improved profile, access to new clients, access to different financing mechanisms). This is consistent with examples from PPP in the Asia Pacific focussed on adult health where private providers had access to free essential medicines and diagnostics, access to training and supervision, and branding to align the provider with the national program and generate demand. [25], [26], [27], [28], [29], [30], [31], [32], [33], [34], [35], [36], [37], [38] This is also consistent with approaches more globally. [39] By contrast, incentives for the public sector were often less well articulated and/ or more altruistic (improved services for adolescents), but identifying these benefits were nonetheless important to ensuring enhanced partnership.

The recommendations should help to strengthen PPP for adolescent health. A key recommendation is that there needs to be clear leadership, however this leadership need not be from government, and perhaps NGOs can play a convening role. Indeed, a lack of leadership and unclear inter-institutional arrangement are recognised barriers to ineffective PPP. [40] In most published examples for adult-focussed PPP in the Asia Pacific region, the government had a lead role in coordination and management of the partnership, with core responsibilities specified at national, district and local level. However, there were examples of coordination being provided by NGOs acting as intermediaries between the public and private sectors, drawing on their greater management expertise and capacity. [25,31,33,[41], [42], [43]]

A key motivation for public private partnership is to improve adolescent health outcomes in the Asia Pacific region. Participants in this study were optimistic, however it is important to note that evidence of the effectiveness of private sector engagement to improve health outcomes more broadly are limited, particularly for the Asia Pacific region. Several studies have demonstrated that PPPs can improve access, utilisation and possibly quality of services. Much of this evidence comes from initiatives focussed on maternal and child health or tuberculosis that have shown an increased uptake of key interventions (such as TB screening and referral, initiation of treatment, or access to obstetric care). [29,30,33,41,[44], [45], [46], [47]] Similarly, approaches such as contracting and voucher schemes may improve the provision of some health services, access for target populations, and reduction in out of pocket expenses, but the impacts on quality, efficiency and equity are unclear. [48] Some social franchising approaches also reported an increase in availability and uptake of contraception in underserved areas, however others found no improvement in quality or coverage of services. [45,[49], [50], [51], [52], [53]] A 2016 Cochrane review of private for-profit providers in LMIC found that provision of training can improve quality and adherence to recommended practice, and regulation may improve quality (although evidence was weak). [54]

This study has several limitations. We undertook this research in three countries that were interested in implementing enhanced PPP (perhaps because of a history of PPP), and thus our findings may not be generalisable to settings were such interest does not exist. However, this interest in PPP and the strong engagement of stakeholders helps ensure that findings may help catalyse action and investment in these settings; notably all three countries identify PPP in their national health policy, with Myanmar and the Philippines recognising its importance for adolescent health. Further, we were unable to complete the final workshop in Myanmar given the current political situation. We remain committed to continuing to work with our colleagues in Myanmar to support adolescent health services.

This study describes current public private partnership in three Asia Pacific countries and develops clear recommendations for implementing public private partnership for adolescent health. We hope it helps to mobilise the resources and knowledge that exist in the private sector and re-align the relationship between these sectors to enhance service access and quality for all adolescents.

**Box 1: Key recommendations to strengthen public-private partnership for adolescent health**

1. Recognise adolescent health as a priority area for action a. Collect data on the needs of adolescents b. Understand the availability and readiness of key stakeholders for partnership

2. Clearly define goals and incentives for partnership a. Define a goal that aligns with needs and/or policy b. Outline clear incentives for all stakeholders involved

3. Establish a partnership model with clear roles for all stakeholders a. Engage the right stakeholders, including adolescents b. Recognise and utilise the strengths of all stakeholders c. Establish clear leadership (need not be led by government,? NGOs/ UN as convenors) and ensure roles for all d. Develop supportive policy and legislation, but not onerous

4. Ensure coordination of public and private sectors a. Internal i. Clear and regular communication between sectors ii. Integration of data systems across stakeholders (and logistic support) b. External i. Clear communication of partnership ii. Recognition of contribution of all partners

5. Provide training and capacity development in adolescent health for all stakeholders

6. Plan for sustainability a. Workforce b. Financing (including access to insurance) c. Exit plans

7. Monitor and regulate activities

8. Evaluate impacts and communicate these to all stakeholders.

## Contributions of each author

EK, SP and PA conceptualised this study. EK and PA developed the study protocol and data collection tools. PA, SP, BD, MB, PH, and ZS supported local implementation of the study in country, with workshops and interviews led by PA, with ZYS leading interviews in Myanmar. PA and JH led the analysis, with all authors contributing to the interpretation and drafting of the manuscript. All authors approved the final manuscript and were responsible for the decision to submit the manuscript.

## Data sharing

Qualitative transcripts are not available for sharing due to the risk of identifying individuals.

## Funding

UNICEF. (Translated abstracts will be provided)

## Research in context

Evidence before this study

We searched the literature to identify models of private sector engagement in health care provision, challenges, and considerations for policy and implementation in Asia and the Pacific. We used broad search terms to identify any study published between 2010-2019 exploring private sector engagement across low- and middle-income countries in the Asia Pacific, searching Medline (OvidSP), Cinhal (Ebsco Host), Embasae (OvidSP), PubMed, Scopus and PsycINFO in November 2019. We identified 55 studies but none focussing specifically on adolescents (detailed in Appendix 1); one study (Munroe, *Global health, science and practice* 2015) explored social franchising to improve access to contraception and reported that coverage and uptake was lower among adolescents compared with adult women. Most studies focused on reproductive, maternal, newborn and child health (RMNCH) or tuberculosis (TB). Of these available studies there was limited detail on the model of private sector engagement, little discussion of the challenges, and limited detail on implementation, including factors that contributed to successful or unsuccessful approaches. Our synthesis of these studies identified the following broad considerations which may be useful in policy and implementation: clear governance, engagement of partners at all stages, information sharing and feedback, training, and clear incentives for all partners.

Added value of this study

This is the first study to comprehensively explore how the private sector can be better engaged in partnership to address adolescent health within resource constrained settings in the Asia Pacific region. A strength of this study is the breadth of inquiry. We included three countries (Mongolia, Myanmar and the Philippines) all motivated to strengthen private sector engagement for adolescent health and with substantial diversity in adolescent population, adolescent health needs, quality of health services, and investments in the public and private sectors (see Table 1). We also included a broad range of stakeholders (including private and public sector actors and young people) across both workshops and key informant interviews. This study generated rich data around the perceptions of the private sector, perceived quality of public and private sector services, training and regulation around adolescent health, challenges and advantages of public-private partnership (for adolescents, the public sector and private sector) and existing models of partnership. These data were systematically analysed and used to develop recommendations for effective partnership which were validated and refined at a further round of participatory workshops. These findings can help re-align the relationship between these sectors to enhance service access and quality for adolescents.

Implications of all the available evidence

These findings fill a significant knowledge gap. While there have been calls at the global and regional levels to engage the private sector in adolescent health, there has been little evidence to guide policy and implementation. We identified a genuine willingness across stakeholders for stronger public-private partnership (PPP), recognising the potential for improved adolescent health service accessibility and quality. Stronger partnership was considered to benefit the public sector by reducing the burden on services and introducing new technologies, while for the private sector advantages included improved access to training and resources, and an enhanced public image. Strengthened PPP was identified to play a particular important role in addressing emerging health issues, sensitive issues, and playing a stronger role in preventative and promotive interventions to complement the public sector's focus on therapeutic care. Key recommendations included the need to establish the foundations for partnership, definition of roles and co-ordination of sectors, ensuring capacity and sustainability, and monitoring and evaluation. These findings define a way forward for improving private sector engagement, and developing more responsive models of service-delivery to ensure equitable access for adolescents.

## Declaration of Competing interest

The authors declare no conflict of interests.

## Appendix Supplementary materials

Image, application 1
