# Supplementary material for: Key recommendations to strengthen public-private partnership for adolescent health in resource constrained settings: Formative qualitative inquiry in Mongolia, Myanmar and the Philippines
Source: Lancet Reg Health West Pac. 2021 Aug 5;15:100242. doi: 10.1016/j.lanwpc.2021.100242 (PMC8357832; doi:10.1016/j.lanwpc.2021.100242)
Supplement: Supplementary file 1 [file mmc1.docx]

# Appendix 1: Summary of literature review

We searched the literature to identify models of private sector engagement in health care provision, challenges, and considerations for policy and implementation in Asia and the Pacific. We used broad search terms to identify any study published between 2010-2019 exploring private sector engagement across low- and middle-income countries in the Asia Pacific, searching Medline (OvidSP), Cinhal (Ebsco Host), Embasae (OvidSP), PubMed, Scopus and PsycINFO in November 2019. The specific search terms were:

((("private sector" OR "private provider" OR "public-private" OR "informal sector" OR "informal provider" OR "traditional provider" OR "traditional healer" OR non-government) AND ( "2015/01/01"[PDat] : "2019/12/31"[PDat] ))) AND (((((((((((((((((((((((((((((((((((((((Asia) OR Pacific) OR Oceania) OR "South Asia") OR "East Asia") OR "South-east Asia") OR Fiji) OR Kiribati) OR "Marshall Islands") OR Micronesia) OR Nauru) OR "Papua New Guinea") OR Samoa) OR "Solomon Islands") OR Tonga) OR Tuvalu) OR Vanuatu) OR Cambodia) OR Indonesia) OR Laos) OR "Lao PDR") OR Malaysia) OR Myanmar) OR Philippines) OR Thailand) OR Timor-Leste) OR Vietnam) OR China) OR Korea) OR Mongolia) OR Afghanistan) OR Bangladesh) OR Bhutan) OR India) OR Iran) OR Maldives) OR Nepal) OR Pakistan) OR "Sri Lanka")

We identified 55 studies but none focussing specifically on adolescents; one study (Munroe, *Global health, science and practice* 2015) explored social franchising to improve access to contraception and reported that coverage and uptake was lower among adolescents compared with adult women. Most studies focused on reproductive, maternal, newborn and child health (RMNCH) or tuberculosis (TB) as detailed in the accompanying table:

| **Author** | **Year** | **Country** | **Focus of PPP model** |
| --- | --- | --- | --- |
| Amarasinghe | 2018 | Asia Pacific | RMNCH |
| Ananthakrishnan | 2019 | India | TB |
| Aung | 2017 | Myanmar | RMNCH |
| Azmat | 2018 | Pakistan | RMNCH |
| Azmat | 2016 | Pakistan | RMNCH |
| Baliga | 2016 | India | RMNCH |
| Bennett | 2017 | Asia Pacific | Malaria |
| Bishai | 2015 | Myanmar | RMNCH |
| Boddam-Whetham | 2016 | Pakistan | RMNCH |
| Bresee | 2018 | Asia Pacific | Influenza |
| Chadha | 2018 | India | TB |
| Daftary | 2019 | India | TB |
| Datta | 2019 | India | TB |
| Deo | 2019 | India | TB |
| Douglass | 2015 | India | Emergency medicine |
| Drewnowski | 2018 | Asia Pacific | Malnutrition |
| Dutta | 2018 | India | General |
| Fernando | 2018 | Sri Lanka | Malaria |
| Field | 2018 | PNG | General |
| Haemmerli | 2018 | India | RMNCH |
| Hemavarneshwari | 2019 | India | TB |
| Herberholz | 2015 | Asia | General |
| Imtiaz | 2017 | Pakistan | RMNCH |
| Iyer | 2016 | India | RMNCH |
| Khan | 2015 | Asia | TB |
| Lei | 2015 | Asia Pacific | TB |
| Lestari | 2017 | Indonesia | TB |
| Mahaarani | 2017 | Indonesia | General |
| Mburu | 2016 | Myanmar | HIV |
| Miller | 2018 | India | General |
| Minh | 2013 | Viet Nam | General |
| Morad | 2015 | Asia | NCD |
| Munroe | 2015 | Asia | RMNCH |
| Nachtnebel | 2015 | Asia Pacific | General |
| Nair | 2019 | India | TB |
| New | 2017 | Myanmar | TB |
| Phok | 2017 | Asia | Malaria |
| Reviono | 2017 | Indonesia | TB |
| Riaz | 2015 | Pakistan | RMNCH |
| Saha | 2018 | India | TB |
| Salazar | 2019 | India | RMNCH |
| Salve | 2018 | India | TB |
| Salve | 2016 | India | TB |
| Sembiah | 2018 | India | General |
| Simmalavong | 2017 | Lao PDR | Malaria |
| Sriram | 2017 | India | Emergency medicine |
| Thet Lwin | 2017 | Myanmar | TB |
| Thiessen | 2018 | PNG | RMNCH |
| Thurston | 2015 | Asia | RMNCH |
| Tougher | 2018 | India | RMNCH |
| Waldrop | 2018 | India | HIV |
| Wang | 2019 | China | General |
| White | 2016 | Lao PDR | RMNCH |
| Yadav | 2017 | India | RMNCH |
| Yasobant | 2016 | India | RMNCH |

# Appendix 2: Workshops

In each country we first held a workshop to introduce the project, seek stakeholder buy-in, identify the key private sector actors and also stakeholders for the interviews. We also used the workshops to explore key concepts of public- private partnership to inform subsequent interviews

A broad range of stakeholders were invited, including government, health care providers from both the public and private sectors, NGOs, relevant UN agencies, faith-based organisation, and youth advocates. The whole day workshop began with an introduction to the project and an overview of existing knowledge around private sector engagement. This included a summary of the available literature for the region (presented in Appendix 1), a discussion around the formal definition of the private sector (non-government entities including both for profit and not for profit, and formal and informal providers), as well as different models for public-private partnership (formal public-private partnership to achieve a joint objective, contracting in or out, social marketing/ franchising, and supporting education and training through private education institutions).

There were then 4 group discussions and feedback sessions exploring: existing private sector engagement in relation to health; private sector engagement specific to adolescent health and lessons learned; advantages and challenges of private sector engagement; and key knowledge gaps for strengthening private sector engagement. Workshops were held in Mongolia (Ulaanbaatar) on the 19^th^ of November 2019 and attended by 43 participants, Philippines (Manilla) on the 11^th^ February 2020 and attended by 34, and Myanmar (Nay Pyi Taw) on the 17^th^ February 2020 and attended by 41.

# Appendix 3: Key informant interview question guide

| **Theme** | **Questions** |
| --- | --- |
| Adolescent care-seeking behaviour and preferences | Where do adolescents and young people (10-24 years) seek care for health problems or advice?  What non-public or private entities do adolescents seek health services from? Do these differ depending on the health issue (SRH, mental health, drug or alcohol, general health problem)?  Why might adolescents seek services from private providers? |
| Definition, composition and roles of the private sector | What does the ‘private sector’ mean in the context of adolescent health?  What types of private sector entities have been involved in delivery of health services to adolescents?  What are their roles / functions in delivery of health services to adolescents?  What private sector entities *should* be involved in delivery of health services to adolescents, and what should their roles be?  What services do private providers deliver to adolescents?  What is your opinion about the quality of these services? Why do you say that?  Which groups of adolescents do they serve (married, unmarried, school-based, work-based, marginalised, etc)?  Where are private providers typically located (type of facility, urban/rural)? |
| Governance, regulation and training | What training does the private sector receive with respect to adolescent health? Who provides this? Do private providers have access to government training?  What training do you think private providers need?  What regulations, legislation or policies exist that relate to private health providers? Are you aware of any regulations specifically related to adolescent health?  To what extent are private entities aware of, or supportive of, government adolescent health policies, guidelines and programs? |
| Advantages of the private sector | What benefits or added value does the private sector bring to adolescent health services?  What particular skills, expertise, technologies does the private sector bring to adolescent health?  What particular aspects of adolescent health could the private sector contribute to?  What are the potential benefits of engaging the private sector in adolescent health, for adolescents, government, UN agencies?  What advantages are there for the private sector to become more involved in adolescent health? What advantages might there be for the private sector to engage in partnership with government and/or UN agencies, NGOs? |
| Challenges and considerations | What are some of the key challenges when engaging the private sector in adolescent health?  What resources or supports to private providers need to engage effectively in adolescent health?  What government mechanisms are needed for effective engagement (regulation, governance, financing, accreditation, etc)?  Are there any disadvantages to engaging the private sector in adolescent health? |
| Models of private sector engagement | What are some effective models of engaging the private sector for adolescent health that you are familiar with (contracting, public-private partnerships, social franchising, etc)? What made these successful?  What might be some effective models of engagement? Why?  Are there any examples of private sector engagement outside of adolescent health that you are aware of that could be transferred to adolescent health?  What do you think would be the key steps needed to more effectively engage the private sector in adolescent health? |
| Any other issues? | Are there any other issues you would like to raise that we have not yet covered today?  I will go over a summary of what we have discussed, if you would like to add to or change anything you have said please let me know |
